# *Listeria innocua* isolated from diseased ruminants harbour minor virulence genes of *L. monocytogenes*

**DOI:** 10.1002/vms3.710

**Published:** 2022-01-18

**Authors:** Carolina Matto, Bruno D'Alessandro, María Inés Mota, Valeria Braga, Alejandro Buschiazzo, Edgardo Gianneechini, Gustavo Varela, Rodolfo Rivero

**Affiliations:** ^1^ Laboratorio Regional Noroeste DILAVE ‘Miguel C. Rubino’ DGSG‐MGAP Paysandú Uruguay; ^2^ Departamento de Desarrollo Biotecnológico Instituto de Higiene, Facultad de Medicina Universidad de la República Montevideo Uruguay; ^3^ Departamento de Bacteriología y Virología Instituto de Higiene, Facultad de Medicina Universidad de la República Montevideo Uruguay; ^4^ Laboratorio de Microbiología Molecular y Estructural Institut Pasteur de Montevideo Montevideo Uruguay

**Keywords:** cattle, genome, listeriosis, sheep, virulence factors

## Abstract

Listeriosis is one of the most common nervous diseases in ruminants, and is caused almost exclusively by the Gram‐positive bacterium, *Listeria monocytogenes*. However, there are few reports of listeriosis associated with *L. innocua*, which is genetically closely related to *L. monocytogenes*, but considered non‐pathogenic. In this work, we report two cases of suppurative meningoencephalitis in apparently previously healthy ruminants from different farms, in which two strains of *L. innocua* were recovered. The whole genomes from both isolates were sequenced, allowing phylogenetic analyses to be performed, which indicated that the two strains were very closely related. Virulence determinants were searched, especially genes coding for the main *L. monocytogenes* virulence factors which have been previously described in *L. innocua*. Surprisingly, the two isolates do not possess such virulence determinants. Instead, both strains carried a set of genes that encode for other virulence factors of the genus *Listeria* detected  using the Virulence Factor Database (VFDB): *iap* (division and invasion of host cells), *lpeA* (entry into non‐professional phagocytes cells), *fbpA* (multifunctional virulence factor, including adherence to host cells)*, lspA* (surface protein anchoring), *lap* (adhesion to enterocytes and trans epithelial translocation)*, pdgA* (resistance to lysozyme), *oatA* (resistance to different antimicrobial compounds and also required for growth inside macrophages)*, lplA1* (use of host‐metabolites for in vivo growth), *gtcA* (catalyses teichoic acid of bacterial wall), *prsA2* (cell invasion, vacuole lysis and intracellular growth), *clpC, clpE* and *clpP* (survival under several stress conditions). These genes among others detected, could be involved in the ability of *L. innocua* to produce damage in animal and human hosts. These results highlight the multifactorial profile of *Listeria* pathogenesis and the need for comprehensive scientific research that address microbiological, environmental and veterinary aspects of listeriosis.

## Abstract 2

This paper report two cases of nervous listeriosis in ruminants due to *L. innocua* in which their genomes were sequenced, and the presence of virulence factors were studied.

## 1 INTRODUCTION

Listeriosis is one of the most common nervous diseases reported in ruminants. The etiological agent responsible for the disease is *Listeria monocytogenes*, a Gram‐positive, facultative intracellular bacterium, which enters the host through contaminated feed (Walland et al., 2015).

*L. innocua* is genetically close to *L. monocytogenes*, but classically considered non‐pathogenic (Buchrieser et al., 2003). It has a wide distribution in the environment, including in ruminant farms as well as in the food industries (Matto et al., 2018; Moreno et al., 2012). Contradicting the classical idea, it has been shown that haemolytic strains of *L. innocua* are capable of infecting eukaryotic cells in experimental models like mouse and zebrafish (Moura et al., 2019). However scarce, there are reports of this agent in humans (Favaro et al., 2014; Perrin et al., 2003) and animal infections (Rocha et al., 2013; Walker et al., 1994).

Here we describe two cases of nervous listeriosis in ruminants associated with non‐haemolytic *L. innocua* isolates, as well as the phylogenetic analysis and the virulence profile based on the complete genomic sequences.

## 2 MATERIALS AND METHODS

The Northwest Regional Laboratory of DILAVE ‘Miguel C. Rubino’, DGSG‐MGAP Uruguay, received the complete central nervous system (CNS) (cerebral hemispheres, cerebellum and brainstem) of a 1‐year‐old Aberdeen Angus bull. The animal came from a beef cattle operation in Flores County, Uruguay. The bull showed circling, aggressiveness and ataxia. The clinical symptoms lasted two days until death. Of 41 bulls in the herd, only one was affected.

A second specimen was received from a different farm, corresponding to the whole CNS of a 1‐year‐old crossbreed sheep. The animal showed lateral left head deviation and circling. The nervous symptoms lasted seven days until death. It was the only sheep affected from a flock of 120 animals, located in Paysandú County, Uruguay.

The CNS of both animals was sectioned longitudinally in two halves. One half was fixed in 10% buffered formalin for histopathology. Different anatomical sections were dehydrated, embedded in paraffin and cut at 5 μm for haematoxylin and eosin staining.

The other half was immersed in buffered Listeria enrichment broth (BLEB) (Oxoid®) and incubated for 48 h at 30°C in aerobiosis. At 24 and 48 h, 100 μl of broth was scattered on a Modified Oxford Agar plate (MOX) (Oxoid®). Plates were incubated in aerobiosis at 35°C, checking for growth at 24 and 48 h. Small white colonies surrounded by a black halo, were transferred to a 5% sheep blood agar plate (SBA‐5%) and also, inoculated in a 1.5 ml vial with tripticase soybean broth (TSB) plus glycerol to preserve them at –80°C. To identify the isolates, we performed Gram stain; catalase test, esculin hydrolysis; and sucrose, glycerol, D‐xylose, D‐mannitol and D‐mannose utilisation assays (Carlin et al., 2021; Hitchins et al., 2020). We also used the API *Listeria* system (BioMérieux®) following the manufacturer's instructions. *L. monocytogenes* ATCC 19111 was included as control. PCR was also performed to detect the *inlA* gene, according to Liu et al. (2007).

Genomic DNA from both isolates was extracted with the DNeasy Blood & Tissue kit (Qiagen®) and used for whole‐genome sequencing (WGS) on an Illumina MiSeq platform with a TruSeq Nano library kit. The reads where trimmed using the software Trimmomatic (version 0.39) (Bolger et al., 2014), with the following parameters ILLUMINACLIP: ‘adapter file’:2:30:10 LEADING:20 TRAILING:20 SLIDINGWINDOW:5:20 AVGQUAL:20 MINLEN:90, being that the adapter file used (TruSeq3‐PE‐2.fa) is part of the Trimmomatic distribution. Genome assembly was performed using SPAdes software (version 3.13.1) (Prjibelski et al., 2020) with the following settings: ‐k 21,33,55,77 –careful –only‐assembler –cov‐cutoff ‘auto’. Quality of assemblies was assessed using QUAST software (version: 5.0.2) (Gurevich et al., 2013) (see Supplementary Table S1). Genome‐based species identification was performed using the average nucleotide identity (ANIb) with the reference genome Clip11262 (NCBI accession NC_003212.1) (http://enve‐omics.ce.gatech.edu/ani/) (Rodriguez‐R & Konstantinidis, 2014).

The genomes of other 12 *L. innocua* strains were selected from the NCBI Genome Database (https://www.ncbi.nlm.nih.gov/genome/) for phylogenetic and virulence profile analysis (see Supplementary Table S2). Whole genome phylogeny was performed from the assemblies using the Enterobase Tool Kit (EToKi) pipeline (Zhou et al., 2020). First, the *align* module was run using the genome sequence of strain Clip11262 as the reference with the following options: ‐a‐ and ‐c 1. Then, the alignment output file obtained in the previous step was used to perform the phylogeny using the RAxML‐NG software (version 0.9.0) (Kozlov et al., 2019) with the following options: –model GTR+G, –seed 3 and –bs‐metric fbp. Using this setup the bootstrapping converged after 550 replicates. The tree support values were drawn on the best‐scoring tree (best value for estimated likelihood).

To complement this information, a comparative test was performed to count the difference in single nucleotide polymorphisms (SNPs) between the two isolates. To obtain specific SNPs between pairs of genomes, the *nucmer* module of the MUMmer software (Marçais et al., 2018) (version 4.0.0) was used with the settings –maxmatch and ‐l 12, followed by the *show‐snps* module with the –CHlrT setting.

Finally, genome sequence analysis was focused on the presence of genes encoding putative virulence factors. The software ABRicate version 1.0.1 (https://github.com/tseemann/abricate) was run with default parameters (≥80% sequence identity and  ≥80% sequence coverage) using the core dataset from the Virulence Factor Database 2.0 (VFDB) (last update in April 2020). The core dataset of VFDB only contains genes from virulence factors experimentally verified from the same genus as the query genome (Liu et al., 2019). For all the genes found using this approach, the presence of premature stop codons and partial sequencing coverage was checked by manual curation. To search for orthologs of these putative virulence genes, the genomes were first annotated using Prokka (Seeman, 2014), to then retrieve target genes’ protein sequences from the .faa files, together with their genome feature metadata (.gff files). Automatic annotations were confirmed by finding orthologous sequences on the UniProtKB database with two rounds of PSI‐BLASTp (blast.ncbi.nlm.nih.gov) followed by multiple sequence alignments of hits (using Cobalt at the NCBI server), spanning different *Listeria* species and other bacterial genera including pathogenic species. These results also allowed us to manually confirm conservation of key residues of target proteins in some cases. A more extensive virulence profile of all the *L. innocua* genomes used in this work was also performed using the BIGSdb‐*Lm* server from the Institut Pasteur (Moura et al., 2016); however, these results were not manually curated. The plugin Gene Presence was ran using the Virulence scheme and the following parameters: ≥80% sequence identity, ≥80% sequence coverage and 20 bp BLASTN word size (https://bigsdb.pasteur.fr/cgi‐bin/bigsdb/bigsdb.pl?db=pubmlst_listeria_isolates).

## 3 RESULTS

Histopathology analyses of both CNS samples showed a moderate suppurative meningoencephalitis with presence of multifocal microabscess lesions, compatible with listeriosis.

In both CNS cultures grew suspicious colonies of *Listeria* spp. The colonies were non‐haemolytic, made up of Gram‐positive rods, catalase‐positive, capable of hydrolysing esculin, sucrose and glycerol fermenters, and unable to produce acid from D‐xylose, D‐mannitol or D‐mannose. The API Listeria yielded the bionumber 7510 corresponding to *L. innocua* with a 99.6% probability. They were PCR‐negative for the gene encoding internalin A. According to these results, the isolates were identified as *L. innocua*, and named *L. innocua* 1074 (cattle isolate) and *L. innocua* 1174 (sheep isolate), respectively.

Whole genomes from both isolates were sequenced (reads are available in the SRA database from NCBI; see supplementary Table S2), and their ANIb analysis confirmed both as *L. innocua* species (98.8% identity with the reference genome).

**FIGURE 1:**
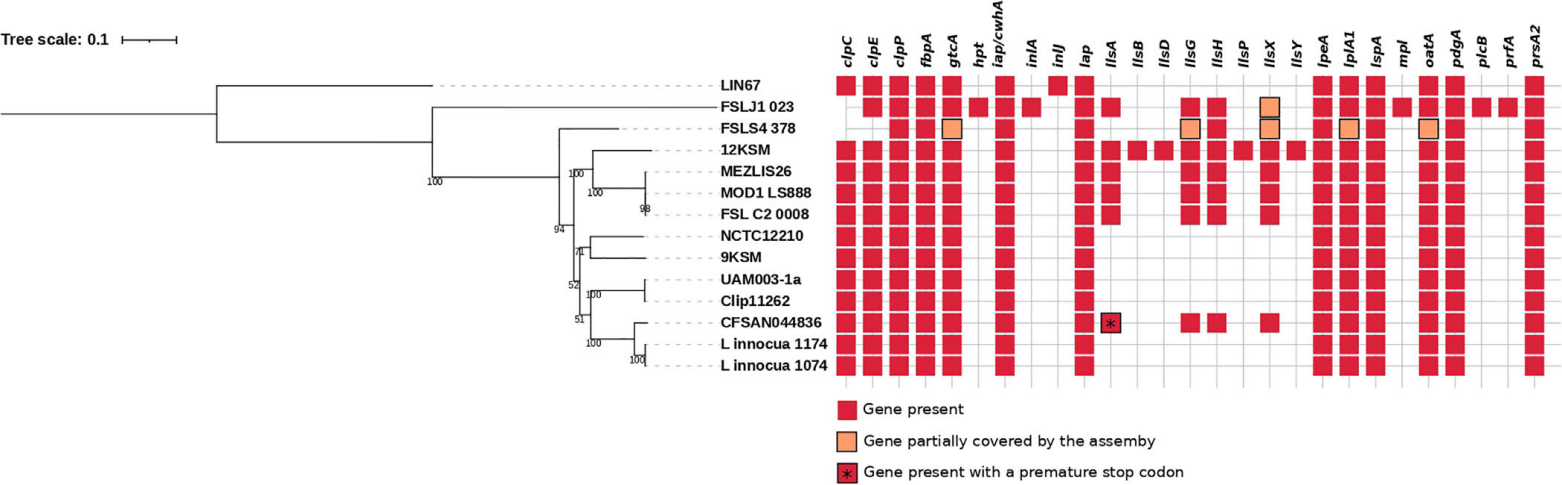
Whole genome maximum likelihood phylogenetic tree including the two reported *L. innocua* strains (*L innocua* 1074 and *L innocua* 1174) and another 12 representative strains of the *L. innocua* diversity, selected from the NCBI Genome Database. The tree is rooted to the LIN67 leaf and the reference genome is Clip11262. Coloured boxes represent the presence of the different genetic traits obtained with the VFDB core dataset. Red boxes indicate the presence of a complete gene. Red boxes with stars indicate truncated genes due to the presence of internal stop codons. Pale orange boxes indicate genes partially covered by the assembly. FBP bootstrap values are shown

A whole genome phylogenetic analysis was performed, including the two isolates reported here and 12 additional genomes that span *L. innocua* diversity. The obtained tree showed that the local isolates obtained from both animals were closely related to each other (Figure 1). This was further studied by a SNPs comparative analysis, which resulted in a difference of 86 SNPs using the cattle isolate as reference, and of 115 SNPs if the reference was the sheep strain.

Virulence factor profiling using the VFDB core dataset identified the presence of 13 genes conserved in both isolates as shown in Figure 1. Manual curation of these results showed that none of these genes had premature stop codons or failed to be covered by the sequencing process. Through this analysis we also found that the virulence factors harboured in these two isolates were also present in most of *L. innocua* reference strains (Figure 1). A more extensive profiling using the BIGSdb‐Lm confirmed these results as well as the presence of other genes potentially involved in *Listeria* pathogenesis, which were also present in the other *L. innocua* genomes used in this study (see Supplementary Table S3).

## 4 DISCUSSION

In this work *L. innocua* was isolated from the central nervous system of two animals with encephalitis. Along with the observed histopathologic lesion in the CNS, this supports the hypothesis that *L. innocua* was the agent responsible for the nervous symptoms of these animals. It has been previously described that cattle are asymptomatic carriers of *L. innocua*, shedding this microorganism in the faeces (Hofer & Reis, 2005; Matto et al., 2018). However, there are only two reports of *L. innocua* as the cause of meningoencephalitis in ruminants (Rocha et al., 2013; Walker et al., 1994), to be contrasted to the numerous reports of animal listeriosis due to *L. monocytogenes* strains (Walland et al., 2015). This is the first report of nervous listeriosis in ruminants due to *L. innocua* in which their genomes were sequenced, and the presence of virulence factors was studied.

The narrow phylogenetic difference found between the genomes of the two isolates is quite interesting (Figure 1), as they were isolated from different animals in two unrelated farms, distanced more than 150 km apart, and without exchange of animals between them. This result suggests a common source of contamination or, a common circulation pathway for these bacteria in ruminants throughout the country or the region. Also, our results encourage the need to maintain the surveillance of cases of nervous diseases in ruminants, and to sequence more *Listeria* isolates, in order to confirm or refute these hypotheses.

Neither of both isolates showed ß‐haemolysis in SBA‐5% plates. This finding is consistent with the absence of the *hly* gene, coding for haemolysin O (a cholesterol‐dependent pore‐forming toxin), one of the main virulence factors of *L. monocytogenes*, located in the cluster called LIPI‐1 (Vázquez‐Boland et al., 2001). The absence of *hly* motivated us to search for the entire LIPI‐1 cluster, finding that neither of both isolates possessed any LIPI‐1 genes. Other authors have reported ß‐haemolytic *L. innocua* strains, but harbouring LIPI‐1 genes (such as *prfA*, *plcA*, *hly*, *actA*, *mpl* and *PlcB*) (Clayton et al., 2013; Johnson et al., 2004; Moura et al., 2019). Similarly, Moreno et al. (2012) described six *L. innocua* isolates with the presence of the *hly* gene and the *inlAB* operon.

Moving further in the study of the genetic background of these *L. innocua* isolates, we did not find any of the internalin genes. The absence of the *inlA* gene was first detected by PCR and then confirmed with the whole genome sequences. Additionally, neither of these two isolates possess the listeriolysin S gene cluster (LIPI‐3) of *L. monocytogenes*. Other reports described the presence of *inlA* gene in 9 out of 42 *L. innocua* isolates (Moura et al., 2019), or the LIPI‐3 cluster (Clayton et al., 2013; Moura et al., 2019). However, both isolates harboured full LIPI‐4 cluster and involved in placental and neural tropism of *L. monocytogenes* clonal complex CC4 (Maury et al., 2016). Moura et al. (2019) also reported the presence of the LIPI‐4 cluster in most of the *L. innocua* isolates studied in their work; however, some of them presented mutations.

Taking into account our results, we wonder if the genes identified in VFDB (Figure 1) could provide the molecular basis to explain the pathogenic behaviour of the isolates of this work. Some of these genes have been reported in *L. monocytogenes* and other Gram‐positive bacteria, individually associated with roles in virulence and/or pathogenicity (Burkholder & Bhunia, 2010; Forster et al., 2011; Keeney et al., 2007; Meireles et al., 2020; Osanai et al., 2013; Rae et al., 2011; Réglier‐Poupet et al., 2003a,b; Vázquez‐Boland et al., 2001). At least 5 of the 13 virulence genes found encode proteins related to bacterial adhesion to, and/or invasion into mammalian cells. (i) The *iap* gene (*i*nvasion‐*a*ssociated *p*rotein) codes for the extracellular protein p60 with murein hydrolase activity and necessary for bacterial division and invasion of host cells (Vázquez‐Boland et al., 2001; Wuenscher et al., 1993). (ii) The *lpeA* gene encodes a protein that belongs to the Lipoprotein Receptor‐associated Antigen I (LraI) superfamily. LraI proteins are associated to the bacterial surface and include several adhesion proteins from many Gram‐positive pathogenic bacteria such as PsaA adhesins from *Streptococcus pneumoniae*, FimA from *S. parasanguis* and EfmA from *Enterococcus faecium*, among others. Like the other LraI proteins, *L. innocua* LpeA includes an extracelullar SBP domain (‘Streptococcal solute‐binding proteins’), which in *L. monocytogenes* binds Zn^+2^ and Mn^+2^ and mediates the entry to eukaryotic cells including hepatocytes and macrophages (Réglier‐Poupet et al., 2003b). (iii) The identification of the *lspA* gene further confirms the role of LpeA in promoting cell entry. LpsA is a type II signal peptidase, shown to be essential for LpeA maturation (Réglier‐Poupet et al., 2003a), with genetic defects in *lpsA* inducing faulty LpeA maturation, consequent loss of its proper surface localisation, and ultimately significant attenuation of *L. monocytogenes* virulence (Réglier‐Poupet et al., 2003a). (iv) *lap* (*L*isteria *a*dhesion *p*rotein) promotes adhesion to intestinal epithelial cells and facilitates extraintestinal dissemination of the bacteria (Burkholder & Bhunia, 2010). (v) Finally, the *fbpA* gene encodes an adhesin comprising fibronectin‐binding domains, FbpA, that functions as an adhesion protein to host cells, especially hepatocytes (Osanai et al., 2013).

Among the virulence‐encoding genes, these *L. innocua* isolates also include genes coding for enzymes that protect bacteria against host defences, or that enhance their survival within the cytosol of infected cells. For example, the *pdgA* and *oatA* genes (peptidoglycan *N*‐deacetylase and *O*‐acetylase, respectively) may be essential to resist the host's lysozyme. Mutants in these two genes result in increase of peptidoglycan's sensitivity to lysozyme inducing *L. monocytogenes* virulence attenuation (Rae et al., 2011). We also found the *lplA1* gene, which encodes a lipoate‐ligase, an enzyme that promotes *Listeria* cytosolic replication within host cells (Keeney et al., 2007).

Other two genes found in these two genomes encode for enzymes that likely play important roles in maintaining the integrity and stability of the bacterial wall in *Listeria*. The *gtcA* gene encodes an enzyme that catalyses teichoic acid glycosylation on *L. monocytogenes* wall. Proper glycosylation mediates key pathogenicity features: the correct anchoring of major surface virulence factors (Ami e InlB); resistance to antimicrobial peptides and decreased susceptibility to antibiotics (Meireles et al., 2020). The second gene present is *prsA2*, which encodes a peptidyl prolyl *cis‐trans* isomerase that assists in correct protein folding. As such, PrsA2 regulates the maturation and secretion of some proprotein virulence factors (such as phospholipase C PC‐PLC) of *L. monocytogenes* (Forster et al., 2011).

Finally, three genes that encode proteases, *clpC, clpE* and *clpP*, were also identified; these are proposed to act as stress response mediators and to assist with intracellular replication (Vázquez‐Boland et al., 2001).

This genetic background might be consistent with virulence retention in both *L. innocua* isolates from clinical cases of listeriosis. The genes identified using VFDB and BIGSdb‐*Lm*  databases, code for virulence factors called minor or accessory but it has been proven that many are capable of promote cell invasion and/or intracellular replication (Burkholder & Bhunia, 2010; Forster et al., 2011; Keeney et al., 2007; Meireles et al., 2020; Osanai et al., 2013; Rae et al., 2011; Réglier‐Poupet et al., 2003a,b;  Vázquez‐Boland et al., 2001). It is noteworthy that genes identified in both isolates are present in most of the reference strains of *L. innocua* from the NCBI genome database. However, none of them were obtained from diseased ruminants. As we mentioned above, there are only two cases of animal listeriosis due to *L. innocua* reported previously, but their genome sequences are not available to compare with those described here.

Regarding that just one of several animals per farm was affected, other issues to be considered in order to attempt to explain the situation are (i) the previous animal health condition and (ii) bacterial exposure load to which these animals were subjected. Disease could be due to unequal exposure to *L. innocua* present in feed or farm environment, presence of debilitating factors in these animals, or both simultaneously. However, until now, individual risk factors for ruminants are poorly understood (Walland et al., 2015). The findings reported in this work highlight the multifactorial nature of the *Listeria* pathogenesis and reinforce the need for detailed scientific research that include microbiological, environmental and veterinary aspects.

## CONFLICT OF INTEREST

The authors declare that there is no conflict of interest.

## ETHICAL STATEMENT

The authors confirm that the ethical policies of the journal, as noted on the journal's autor guidelines page, have been adhered to. No ethical approval was required because the laboratory received samples which correspond to dead animals at farms.

## AUTHOR CONTRIBUTIONS

**Carolina Matto**: funding acquisition; investigation; methodology; resources; visualisation; writing – original draft; writing – review & editing. **Bruno D'Alessandro**: data curation; formal analysis; methodology; resources; software; visualisation; writing – review & editing. **Edgardo Gianneechini**: investigation; methodology; resources; writing – review & editing. **Gustavo Varela**: conceptualisation; formal analysis; investigation; methodology; resources; supervision; visualisation; writing – review & editing. **Rodolfo Rivero**: conceptualisation; formal analysis; methodology; supervision; visualisation; writing – review & editing. **María Inés Mota**: Data curation, Formal analysis, Investigation, Methodology, Resources, Software, Visualization, Writing – review & editing. **Valeria Braga**: Formal analysis, Investigation, Resources, Software, Writing – review & editing. **Alejandro Buschiazzo**: Data curation, Formal analysis, Resources, Writing – review & editing.

### PEER REVIEW

The peer review history for this article is available at https://publons.com/publon/10.1002/vms3.710.

## Supporting information

Tables 1–2

Table 3

## Data Availability

Both Listeria innocua sequences data have been submitted to the GenBank database under accession number SAMN22746544/SRR16633068 and SAMN22746545/SRR16633067.
