# Supplementary material for: Listeria innocua isolated from diseased ruminants harbour minor virulence genes of L. monocytogenes
Source: Vet Med Sci. 2022 Jan 18;8(2):735–40. doi: 10.1002/vms3.710 (PMC8959264; doi:10.1002/vms3.710)
Supplement: Supplementary file 1 — Tables 1–2 [file VMS3-8-735-s002.docx]

**Supplementary Table 1:** Quality indicators of sequences of *L. innocua* reported in this work and their assemblies

| Strain | Sequencing | | | Assembly | |
| --- | --- | --- | --- | --- | --- |
|  | #reads | per base theoretical coverage (L*N/G) | >Q30 % | #contigs | N50 |
| 1074 | 23,708,002 | 1291 | 93.6 | 32 | 1,424,980 |
| 1174 | 22,398,202 | 1202 | 93.7 | 32 | 1,424,980 |

L=read length, N=#reads, G=genome size for 1074 G=2,771,231 and for 1174 G=2,811,837. Genome sizes were obtained from the total number of bases in the assembled sequences reported in the quast results.

**Supplementary Table 2:** NCBI accession, source and origin data for the *L. innocua* genomes used in this work.

| **Strain** | **BioSample/SRA** | **Assembly accession** | **Source** | **Country** |
| --- | --- | --- | --- | --- |
| *L. innocua* 1074† | SAMN22746544/  SRR16633068 | - | animal | Uruguay |
| *L. innocua* 1174† | SAMN22746545/  SRR16633067 | - | animal | Uruguay |
| FSL C2-0008 | SAMN07411324 | GCA_003384075.1 | environmental | undisclosed |
| NCTC12210 | SAMEA104167173 | GCA_900452995.1 | environmental | United Kingdom |
| 12KSM | SAMN03381211 | GCA_000960735.1 | environmental | Austria |
| 9KSM | SAMN03381197 | GCA_000960585.1 | environmental | Austria |
| MOD1_LS888 | SAMN02769797 | GCA_000773035.1 | environmental | USA-FL |
| FSL S4-378 | SAMN01909076 | GCA_000183885.1 | environmental | USA-NY |
| FSL J1-023 | SAMN01909077 | GCA_000183905.1 | undisclosed | undisclosed |
| Clip11262 | SAMEA3138320 | GCA_000195795.1 | food | France |
| MEZLIS26 | SAMN11604718 | GCA_005577055.2 | animal | South Africa |
| LIN67 | SAMN13663825 | GCA_009807615.1 | food | Russia |
| CFSAN044836 | SAMN04417899 | GCA_009648575.1 | food | Italia |
| UAM003-1a | SAMN12356784 | GCA_008016335.1 | animal | USA-CA |

†: *L. innocua* isolates recovered from CNS of animals with nervous symptoms and histopathological lesions consistent with listeriosis.

‡: Not available until publication.

**Supplementary Table 3:** Presence/absence of virulence genes according to the Bigsdb-*Lm* platform (https://bigsdb.pasteur.fr/listeria) in the isolates reported in this work, as well as in the reference strains used for phylogenetic analyses.

Please refer to Excel file identified as Supp 3

*: Genes found in VFDB (core dataset); 0: gene absent; 1: gene present; NA: not available in BIGSdb, assemblies where uploaded for the purpose of this analysis; † premature stop codon; ‡ partially covered by the assembly.
